# Burden of acute and long-term COVID-19: a nationwide study in Bahrain

**DOI:** 10.3389/fpubh.2025.1539453

**Published:** 2025-03-18

**Authors:** Mariam Murad, Stephen L. Atkin, Pearl Wasif, Alwaleed Abdulaziz Behzad, Aamal M. J. Abdulla Husain, Roisin Leahy, Florence Lefebvre d’Hellencourt, Jean Joury, Mohamed Abdel Aziz, Srinivas Rao Valluri, Hammam Haridy, Julia Spinardi, Moe H. Kyaw, Manaf Al-Qahtani

**Affiliations:** ^1^Royal College of Surgeons in Ireland, Al Muharraq, Bahrain; ^2^Salmanyia Medical Complex, Manama, Bahrain; ^3^Primary Health Care Center, Manama, Bahrain; ^4^Royal College of Surgeons in Ireland, Dublin, Ireland; ^5^Pfizer Inc., New York, NY, United States; ^6^Pfizer Gulf FZ LLC, Dubai, United Arab Emirates; ^7^Pfizer Inc., São Paulo, Brazil; ^8^Bahrain Defence Force Royal Medical Services Military Hospital, West Riffa, Bahrain

**Keywords:** SARS-CoV-2, COVID-19, long COVID-19 disease, respiratory disease, pneumonia

## Abstract

**Background:**

Coronavirus disease 2019 (COVID-19) may lead to long-term sequelae. This study aimed to understand the acute and post-acute burden of SARS-CoV-2 infection and to identify high-risk groups for post-COVID-19 conditions (PCC).

**Methods:**

A retrospective observational study of the Bahraini population was conducted between 1 May 2021 and 30 April 2023, utilizing the national administrative database. PCC cases were defined according to WHO guidelines. All COVID-19 cases were confirmed using real-time polymerase chain reaction (PCR).

**Results:**

Of 13,067 COVID-19 cases, 12,022 of them experienced acute COVID-19, and 1,045 of them developed PCC. Individuals with PCC tended to be older women with risk factors and instances of SARS-CoV-2 reinfection. The incidence rates per 100,000 individuals during the Alpha pandemic surge (2020), Delta pandemic surge (2021), and Omicron pandemic surge (2022) were 2.2, 137.2, and 222.5 for acute COVID-19, and 0.27, 10.5, and 19.3, respectively, for PCC cases. The death rates per 100,000 individuals during the Alpha, Delta, and Omicron pandemic surges were 3, 112, and 76, respectively, for acute COVID-19 and 1, 10, and 8, respectively, for PCC. The death rate was highest among those aged 65 and older during the Delta pandemic surge.

**Conclusion:**

These findings suggest the need for a timely national vaccination program prior to new COVID-19 surges to prevent complications related to SARS-CoV-2 infection, particularly in the older adult and in non-older adult individuals with risk factors.

## 1 Introduction

The devastating COVID-19 pandemic, caused by the novel coronavirus SARS-CoV-2, has led to over 763 million infections and more than 6.9 million deaths globally (1). By May 2024, the Kingdom of Bahrain registered 741,854 cases and 1,602 deaths.

Five vaccines against COVID-19 are currently available free of charge for adults (18 years and older) in Bahrain: BBIBP-CorV (Sinopharm; Beijing, China), BNT162b2 (Comirnaty; Pfizer; New York, NY, United States and BioNTech; Mainz, Germany), ChAdOx1-SARS-COV-2/AZD1222 (CoviShield; AstraZeneca/Serum Institute of India, Pune, India), Valneva (Saint-Herblain, France), and Gam-COVID-Vac (Sputnik V; Gamaleya Research Institute of Epidemiology and Microbiology; Moscow, Russia). Both Sinopharm and Comirnaty are authorized for use in populations under the age of 18 years (2).

The success that the Kingdom of Bahrain has observed in its response to COVID-19 can be largely attributed to its vaccination program. The timeline of this program is shown in Supplementary Figure 1. The Bahrain vaccination initiative was supported by the expansion of Bahrain’s already robust routine vaccination infrastructure, which has maintained over 95% coverage for all childhood vaccines over the past 20 years (1). The vaccination program has also expanded to include booster doses for COVID-19, with recent data indicating that over 85% of the eligible population has received at least one booster dose against the virus (2).

Acute COVID-19 is characterized by respiratory symptoms, fever, and gastrointestinal issues (3), which may lead to hospitalization and death. Following the acute phase, individuals can develop post-acute sequelae of SARS-CoV-2 infection (PCC), also known as post-COVID-19 conditions (PCC) or “long COVID-19.” This condition is characterized by the persistence, recurrence, or emergence of a range of symptoms following the initial SARS-CoV-2 infection. However, its definition remains evolving and varies across health agencies worldwide.

The United States Centers for Disease Control and Prevention (US CDC) describes PCC, or long COVID-19, as symptoms that persist or occur at least 4 weeks after the acute illness (4). The World Health Organization (WHO) defines long COVID-19 as typically occurring in adults 3 months after the onset of SARS-CoV-2 infection, with common symptoms including fever, cough, myalgia, headache, fatigue, anosmia, and ageusia (1, 5). Early studies suggest that patients who experienced more severe illness during the acute phase of infection are more likely to develop long-term symptoms, which may persist for weeks, months, or even years. Persistent symptoms may manifest as different phenotypes involving multiple organ systems, complicating the characterization and understanding of the underlying pathogenic mechanisms (6). Current hypotheses regarding PCC or long COVID suggest that persistent viruses, viral antigens, or viral RNA may lead to inflammation, trigger autoimmunity, or induce changes in the microbiome (7).

Although some studies in countries such as the United States of America (USA) and the United Kingdom (UK) have characterized persistent symptoms following acute SARS-CoV-2 infection (with prevalence rates of 6.9% in the USA (8) and 3.1% in the UK (9)), the definition of “long COVID-19” remains a subject of active scientific investigation. Data from diverse populations and geographical regions globally will help better define and characterize this syndrome and accelerate the development of a harmonized case definition (5, 10).

This nationwide retrospective study was conducted to better understand the burden of SARS-CoV-2 infection during and after the acute phase and to identify groups at higher risk of developing persistent symptoms for targeted programs. Furthermore, these data will serve as the evidence base needed to enhance the development of public health policies and the implementation of proven interventions against COVID-19, such as vaccination across age groups.

## 2 Materials and methods

This was a retrospective, observational database study of the Bahraini population conducted between 1 May 2021 and 30 April 2023. For the analysis of acute COVID-19 burden, a cut-off date of 30 April 2022 was chosen for the study. For the analysis of the PCC burden, data from the first year (1 May 2021–30 April 2022) were collected and followed up in the second year (1 May 2022–30 April 2023). This study was conducted as part of Bahrain’s national COVID-19 public health response under the Infectious Diseases Act, Ministry of Health, Bahrain, and was exempt from ethics review as per the National Taskforce for Combatting COVID-19, Bahrain (NTCC-S-0038-2023). The study was conducted in accordance with the protocol, as well as the legal and regulatory requirements of the Kingdom of Bahrain, and the general principles outlined in the International Ethical Guidelines for Biomedical Research Involving Human Subjects (Council for International Organizations of Medical Sciences 2002), the ICH Guideline for Good Clinical Practice, and the Declaration of Helsinki.

The raw data for this study were extracted from administrative databases maintained by the Ministry of Health (MOH) in Bahrain, which included national records of all confirmed SARS-CoV-2 infections, vaccines administered, and clinical records. Throughout the pandemic, Bahrain used PCR to confirm all SARS-CoV-2 infections. The following national databases were available to the study investigators, who performed data linkages using individual pseudo-IDs:

1. National database of COVID-19 cases: Doctors are legally obligated to notify Bahrain’s Ministry of Health (MOH) of all COVID-19 cases under the Infectious Diseases Act. Additionally, all public hospitals must report patient-level information regarding all COVID-19-related hospitalizations to the MOH.
2. MOH/Public Health: All COVID-19 vaccinations administered in Bahrain are recorded under this registry in the National Electronic Health Record (I-SEHA).
3. I-SEHA: A key enabler of “one patient, one health record, “this system provides a comprehensive longitudinal view of Bahraini patients’ journeys throughout the entire public healthcare network, including inpatient admissions, emergency department (ED) visits, outpatient visits, and information on comorbid conditions.
4. The Public Health Database (Genomic Surveillance for SARS-CoV-2) will facilitate the random selection of 600 cases each month based on their national identity numbers, allowing for monitoring of the pandemic situation and the distribution of variants (11).

All data contained within the MOH databases are secured and encrypted.

We created two patient cohorts (acute COVID-19 and PCC) for the study to explore the burden of COVID-19 among Bahraini citizens and long-term residents aged 3 years and older. For the acute COVID-19 cohort, we included individuals who tested positive for SARS-CoV-2 PCR during their initial presentation. Acute COVID-19 disease included COVID-19-related hospitalizations, emergency room visits, primary care visits, deaths, and cases of myocarditis and pericarditis after the onset of COVID-19. COVID-19 patients were categorized based on healthcare settings, such as inpatient, outpatient, or intensive care unit (ICU), within 90 days of their COVID-19 diagnosis (Supplementary Table S1). With these defined study outcomes, we aimed to estimate the burden of acute COVID-19. The criteria for severe COVID-19 cases included those requiring oxygen supplementation or ICU admission at any point from diagnosis through to discharge or death due to COVID-19.

The PCC cohort included individuals who tested positive for SARS-CoV-2 via PCR more than 3 months after the initial presentation, based on the PCC definition according to WHO criteria (12). Supplementary Table S2 lists the medical conditions considered for long-term COVID-19. These conditions were identified using the ICD-10 codes shown in Supplementary Table S3 and were used to assess the burden of post-acute COVID-19 complications.

The data were extracted and stored using Excel files in password-encrypted, cyber-secure platforms. All data were pseudonymized with a unique code assigned to each patient. Each code was generated by the platform Zoho Creator (version 5.26; Zoho Corp.), which was used for the electronic case report form. The following information was extracted from the I-SEHA: 1. Hospitalization records, including diagnosis codes (at discharge), date of admission, length of stay (LOS), and medical comorbidities; 2. Emergency department (ED) records, including diagnosis codes and date of visit; 3. Primary care and clinic records from family medicine practitioners with advanced training in infectious diseases, including diagnosis codes and date(s) of visit(s).

### 2.1 Statistical analysis

Statistical analysis was conducted using RStudio version 2023.12.1 + 402. Descriptive statistics for demographic characteristics and vaccination status were presented separately for the acute and post-acute cohorts. The characteristics associated with acute COVID-19 and PCC were also presented for participants with and without risk factors. Between-group differences were examined using Welch’s two-sample *t*-tests for continuous variables and either chi-squared tests or Fisher’s exact tests for categorical variables. Chi-squared tests were used when the expected cell count was at least five; otherwise, Fisher’s exact tests were used. A *p*-value threshold of <0.05 was selected *a priori* for all analyses.

The outcomes of the three pandemic surges—Alpha (September 2020), Delta (May 2021), and Omicron (BA.1/BA.2, June 2022)—were presented separately for participants in both the acute and post-acute groups. The total number and percentage of participants experiencing each outcome were provided, along with the calculation of 95% confidence intervals for the outcome percentages.

The incidence and mortality rates per 100,000 individuals for acute COVID-19 and PCC were calculated by age groups during periods of COVID-19 pandemic surges. Data from the Bahrain population census served as the denominator for the incidence calculations, using the mid-year population figure for the relevant year. We evaluated healthcare utilization across various settings, including primary care, emergency room (ER), and hospital care, for both acute COVID-19 and PCC.

## 3 Results

A total of 13,067 cases were reviewed, of which 12,022 had acute COVID-19 alone, while 1,045 met the criteria for PCC. Demographic data are shown in Table 1. In the unadjusted analysis comparing individuals with acute COVID-19, those with PCC were older, predominantly female, of Arab descent, and had higher vaccination status, although no differences were noted in the number of vaccine doses administered. Patients with PCC were more likely to have additional risk factors, including cancer, renal disease, cardiovascular disease, diabetes, respiratory disease, hypertension, and neurological disorders, and they exhibited higher levels of obesity (Table 1).

**Table 1:** Demographic characteristics for the acute COVID-19 and PCC populations.

Characteristic	Acute, *N* = 12,022	PCC *N* = 1,045	*p*-value^b^
Age^a^	37.6 (28.8)	50.1 (29.2)	<0.001
Age group			<0.001
3–17 years	3,852 (32%)	173 (17%)	
18–49 years	4,130 (34%)	320 (31%)	
50–64 years	1,415 (12%)	167 (16%)	
65–74 years	494 (4.1%)	88 (8.4%)	
75+ years	2,127 (18%)	297 (28%)	
Unknown	4	0	
Gender			0.010
Female	5,991 (50%)	564 (54%)	
Male	6,031 (50%)	481 (46%)	
Ethnicity			<0.001
Arab	10,741 (90%)	1,004 (96%)	
Asian	1,144 (9.5%)	35 (3.4%)	
Other	114 (1.0%)	3 (0.3%)	
Unknown	23	3	
BMI (kg/m2)	29.6 (7.2)	29.7 (7.3)	0.7
Unknown	10,359	810	
Vaccination status			<0.001
Non-Vaccinated	3,574 (30%)	198 (19%)	
Vaccinated	8,448 (70%)	847 (81%)	
Number of vaccine doses			0.4
0	4 (<0.1%)	0 (0%)	
1	124 (1.5%)	13 (1.5%)	
2	2,130 (25%)	188 (22%)	
3	6,071 (72%)	636 (75%)	
4	122 (1.4%)	10 (1.2%)	
5	1 (<0.1%)	0 (0%)	
Unknown	3,570	198	
Any risk factor^c^ (yes)	4,406 (37%)	650 (62%)	<0.001
Cancer (yes)	139 (1.2%)	24 (2.3%)	0.001
Chronic renal disease (yes)	303 (2.5%)	72 (6.9%)	<0.001
Chronic respiratory disease (yes)	749 (6.2%)	140 (13%)	<0.001
Cardiovascular disease (yes)	2,273 (19%)	380 (36%)	<0.001
Diabetes (yes)	2,077 (17%)	332 (32%)	<0.001
HIV (yes)	0 (0%)	0 (0%)	>0.9
Hypertension (yes)	2,647 (22%)	447 (43%)	<0.001
Liver disease (yes)	70 (0.6%)	7 (0.7%)	0.7
Neurological disorder (yes)	401 (3.3%)	71 (6.8%)	<0.001
Overweight/obesity (yes)	1,108 (9.2%)	165 (16%)	<0.001
Severe mental disorder (yes)	137 (1.1%)	30 (2.9%)	<0.001
Solid organ transplant (yes)	0 (0%)	0 (0%)	>0.9

a Except for the row labeled age, which is reported as Mean (SD), all other rows report *n* (%).

b Welch Two Sample *t*-test; Pearson’s Chi-squared test; Fisher’s exact test. Acute vs. long COVID.

c Any risk factor = any in the list of conditions detailed in the table.

Table 2 shows the incidence rate per 100,000 individuals and the number of acute COVID-19 cases along with long PCC cases by age group from 1 May 2021 to 30 April 2023, in accordance with the COVID-19 pandemic surges that occurred in Bahrain.

**Table 2:** Cases and incidence rate per 100,000 individuals of acute COVID-19 and PCC by age groups: 1 May 2021–30 April 2023.

Acute COVID-19 (*N* = 12,022)					PCC (*N* = 1,045)			
Incidence (cases)					Incidence (cases)			
Age groups	Alpha pandemic surge	Delta pandemic surge	Omicron pandemic surge	Omicron variant	Alpha pandemic surge	Delta pandemic surge	Omicron pandemic surge	Omicron variant
3–17	1.72 (5)	241.48 (704)	216.78 (632)	862.78 (2515)	0 (0)	11.32 (33)	10.29 (30)	37.73 (110)
18–49	1.29 (12)	67.53 (627)	142.92 (1327)	233.07 (2164)	0.11 (1)	4.85 (45)	10.45 (97)	19.06 (177)
50–64	3.19 (6)	120.30 (226)	212.91 (400)	416.78 (783)	0 (0)	9.58 (18)	23.95 (45)	55.36 (104)
65–74	0 (0)	196.75 (81)	383.78 (158)	619.40 (255)	2.43 (1)	14.57 (6)	51.01 (21)	145.74 (60)
75+	58.39 (9)	2407.06 (371)	4807.06 (741)	6526.96 (1006)	12.98 (2)	337.38 (52)	583.92 (90)	992.67 (153)
Total	2.19 (32)	137.18 (2009)	222.47 (3258)	459.07 (6723)	0.27 (4)	10.52 (154)	19.32 (283)	41.24 (604)

The first peak of the Alpha COVID-19 pandemic surge occurred in September 2020; the second peak of the Delta COVID-19 pandemic surge was in May 2021; and the third peak of the Omicron COVID-19 pandemic surge took place in June 2022. Omicron was the most commonly detected variant, with the first variant case reported in December 2021.

Table 2 shows the incidence rate per 100,000 individuals and the number of cases of acute COVID-19 and long PCC categorized by age group from 1 May 2021 to 30 April 2023, according to the COVID-19 pandemic surges that occurred in Bahrain.

The first Alpha COVID-19 pandemic surge occurred in September 2020, followed by the second Delta surge in May 2021, and then the third Omicron surge in June 2022. Omicron became the most commonly detected variant, with the first variant case identified in December 2021. The incidence per 100,000 for acute COVID-19 and PCC is categorized by age group and COVID-19 wave, indicating an increased incidence of both acute COVID-19 and PCC with each successive pandemic surge. For Omicron infections, there was a significant rise in the 65–74 and 75+ age groups for both acute COVID-19 and PCC (Supplementary Figure 2). The number of deaths and mortality rate per 100,000 individuals for acute COVID-19 and PCC by age group from 1 May 2021 to 30 April 2023 is shown in Table 3.

**Table 3:** Number of deaths and mortality rate per 100,000 individuals for acute COVID-19 and PCC by age group: 1 May 2021–30 April 2023.

Acute COVID-19					PCC			
Mortality rate (cases)					Mortality rate (cases)			
Age groups	Alpha pandemic surge	Delta pandemic surge	Omicron pandemic surge	Omicron variant	Alpha pandemic surge	Delta pandemic surge	Omicron pandemic surge	Omicron variant
3–17	0	0	0	0	0	0	0	0
18–49	0	0.43 (4)	0.11 (1)	0	0	0	0	0
50–64	0.53 (1)	3.73 (7)	1.60 (3)	1.06 (2)	0	0.53 (1)	0	1.06 (2)
65–74	2.43 (1)	19.43 (8)	2.43 (1)	9.72 (4)	0	2.43 (1)	0	4.86 (2)
75+	6.49 (1)	603.39 (93)	460.65 (71)	532.02 (82)	6.49 (1)	51.90 (8)	51.90 (8)	142.71 (22)
Total	0.20 (3)	7.65 (112)	5.12 (76)	6.01 (88)	0.07 (1)	0.68 (10)	0.55 (8)	1.78 (26)

The first Alpha COVID-19 pandemic occurred in September 2020, followed by the second peak of the Delta variant in May 2021 and the third peak of the Omicron variant in June 2022. Omicron became the most common variant detected, with the first case reported in December 2021.

The number and incidence of mortality per 100,000 significantly increased in the 75+ age group, rising from the first to subsequent COVID-19 pandemic surges. The acute characteristics of COVID-19, depending on whether the patient had additional risk factors, are shown in Table 4.

**Table 4:** Characteristics of acute COVID-19 in individuals with and without risk factors in the acute group.

Characteristic	Risk Factors	Risk factors	*p*-value^b^
	Yes, *N* = 4,406^a^	No, *N* = 7,616^a^	
Number of episodes^c^			0.2
1	3,818 (87%)	6,677 (88%)	
2	569 (13%)	901 (12%)	
3+	19 (0.4%)	38 (0.5%)	
Emergency department visit (yes)	266 (6.5%)	75 (1.1%)	<0.001
Unknown	307	684	
Primary care visit (yes)	3,818 (93%)	6,866 (99%)	<0.001
Unknown	307	684	
Hospitalization with a COVID-19 diagnosis as the primary diagnosis (yes)	753 (18%)	142 (2.0%)	<0.001
Unknown	307	684	
Hospitalization where COVID-19 is present on admission (yes)	155 (3.8%)	50 (0.7%)	<0.001
Unknown	307	684	
ICU (yes)	133 (3.2%)	24 (0.3%)	<0.001
Unknown	307	684	
Myocarditis (yes)	2 (<0.1%)	1 (<0.1%)	0.6
Unknown	307	684	
Required a ventilator (yes)	141 (3.2%)	22 (0.3%)	<0.001
Unknown	22	20	
Death (yes)^d^	250 (5.7%)	29 (0.4%)	<0.001
Unknown	22	21	
Time until death			0.4
0–3 months	163 (67%)	23 (79%)	
4–6 months	27 (11%)	3 (10%)	
7–12 months	20 (8.2%)	0 (0%)	
13 months+	34 (14%)	3 (10%)	
Unknown	4,162	7,587	
Cardiovascular disease (yes)	117 (2.7%)	25 (0.3%)	<0.001
Respiratory (yes)	128 (2.9%)	31 (0.4%)	<0.001
Liver disease (yes)	7 (0.2%)	0 (0%)	<0.001
Chronic kidney disease (yes)	30 (0.7%)	3 (<0.1%)	<0.001
Neurological (yes)	39 (0.9%)	12 (0.2%)	<0.001
Diabetes (yes)	0 (0%)	0 (0%)	>0.9
Thrombosis (yes)	0 (0%)	0 (0%)	>0.9
Multi-system inflammatory syndrome (yes)	4 (<0.1%)	6 (<0.1%)	>0.9

a *n* (%).

b Pearson’s Chi-squared test; Fisher’s exact test.

c Each new infection confirmed by a positive PCR test for SARS-CoV-2 infection.

d Mortality by age is shown in Table 3.

For those with acute COVID-19 who have additional risk factors, there were significant differences (*p* < 0.001) regarding the frequency of emergency visits, hospitalizations, ICU admissions, the requirement for a ventilator, and death occurring within the first 3 months of presentation. Additionally, there was a lower rate of primary care attendance compared to those with acute COVID-19 without additional risk factors. Vaccine status revealed no differences between acute COVID-19 patients and those with PCC, with no variation in the age-stratified number of vaccine doses administered. However, experiencing more than one reinfection (as shown in Tables 4, 5) was correlated with the development of PCC (*p* < 0.0001), although the rate of reinfection did not differ based on whether the patient had risk factors. The characteristics of the acute COVID-19 group, according to the COVID-19 pandemic surge, are shown in Table 6.

**Table 5:** Characteristics of PCC patients by risk factors.

Characteristic	Risk factors	Risk factors	*p*-value^b^
	Yes, *N* = 650^a^	No, *N* = 395^a^	
Number of episodes			0.2
1	499 (78%)	316 (80%)	
2	141 (22%)	73 (19%)	
3+	3 (0.5%)	5 (1.3%)	
Unknown	7	1	
Emergency department visit (yes)	52 (8.4%)	7 (1.9%)	<0.001
Unknown	32	28	
Primary care visits (yes)	589 (95%)	363 (99%)	0.002
Unknown	32	28	
Hospitalization with a COVID-19 diagnosis as the primary diagnosis (yes)	107 (17%)	25 (6.8%)	<0.001
Unknown	32	28	
Hospitalization where COVID-19 is present on admission (yes)	27 (4.4%)	6 (1.6%)	0.021
Unknown	32	28	
ICU (yes)	19 (3.1%)	3 (0.8%)	0.020
Unknown	32	28	
Myocarditis (yes)	0 (0%)	1 (0.3%)	0.4
Unknown	32	28	
Required a ventilator (yes)	12 (1.8%)	2 (0.5%)	0.068
Unknown	1	1	
Death (yes)	42 (6.5%)	3 (0.8%)	<0.001
Unknown	0	1	
Time until death			0.7
0–3 months	4 (9.5%)	0 (0%)	
4–6 months	8 (19%)	1 (33%)	
7–12 months	21 (50%)	1 (33%)	
13 months+	9 (21%)	1 (33%)	
Unknown	608	392	

a *n* (%).

b Fisher’s exact test; Pearson’s Chi-squared test.

**Table 6:** Characteristics of the acute COVID-19 group according to COVID-19 pandemic surge.

Acute COVID group	First Alpha pandemic surge		Second Delta pandemic surge		Third Omicron pandemic surge		Omicron variant	
Characteristic	*N* = 732^a^	95% CI^b^	N = 2,381^a^	95% CI^b^	*N* = 3,232^a^	95% CI^b^	N = 7,108^a^	95% CI^b^
Emergency department visit (yes)	36 (5.2%)	3.7, 7.2%	112 (5.1%)	4.3, 6.2%	129 (4.4%)	3.7, 5.2%	113 (1.7%)	1.4, 2.1%
Unknown	41		205		273		582	
Primary care visit (yes)	660 (96%)	94, 97%	2,060 (95%)	94, 96%	2,834 (96%)	95, 96%	6,405 (98%)	98, 98%
Unknown	41		205		273		582	
Hospitalization with a COVID-19 diagnosis as the primary diagnosis (yes)	149 (22%)	19, 25%	262 (12%)	11, 13%	345 (12%)	11, 13%	340 (5.2%)	4.7, 5.8%
Unknown	41		205		273		582	
Hospitalization where COVID-19 is present on admission (yes)	31 (4.5%)	3.1, 6.4%	62 (2.8%)	2.2, 3.7%	73 (2.5%)	2.0, 3.1%	81 (1.2%)	0.99, 1.5%
Unknown	41		205		273		582	
ICU (yes)	14 (2.0%)	1.2, 3.5%	72 (3.3%)	2.6, 4.2%	37 (1.3%)	0.89, 1.7%	46 (0.7%)	0.52, 0.95%
Unknown	41		205		273		582	
Myocarditis (yes)	0 (0%)	0.00, 0.69%	1 (<0.1%)	0.00, 0.30%	0 (0%)	0.00, 0.16%	2 (<0.1%)	0.01, 0.12%
Unknown	41		205		273		582	
Required a ventilator (yes)	4 (0.6%)	0.18, 1.5%	66 (2.8%)	2.2, 3.6%	53 (1.6%)	1.2, 2.2%	45 (0.6%)	0.47, 0.86%
Unknown	5		12		14		19	
Death (yes)	8 (1.1%)	0.51, 2.2%	114 (4.8%)	4.0, 5.8%	78 (2.4%)	1.9, 3.0%	91 (1.3%)	1.0, 1.6%
Unknown	6		11		15		20	
Time until death								
0–3 months	6 (75%)	36, 96%	90 (80%)	72, 87%	55 (72%)	61, 82%	43 (48%)	38, 59%
4–6 months	2 (25%)	4.5, 64%	5 (4.5%)	1.7, 11%	11 (14%)	7.8, 25%	14 (16%)	9.2, 25%
7–12 months	0 (0%)	0.00, 40%	2 (1.8%)	0.31, 6.9%	5 (6.6%)	2.4, 15%	14 (16%)	9.2, 25%
13 months+	0 (0%)	0.00, 40%	15 (13%)	7.9, 21%	5 (6.6%)	2.4, 15%	18 (20%)	13, 30%
Unknown	724		2,269		3,156		7,019	

a *n* (%).

b CI, confidence interval.

The COVID-19 pandemic surges affecting emergency medicine and primary care visits showed little difference; however, there were significantly more hospitalizations during the first COVID-19 pandemic surge compared to the subsequent two surges: 22% (95% CI: 19, 25) versus 12% (95% CI: 1, 13) and 12% (95% CI: 11, 13), respectively. Additionally, the second surge had more ICU admissions than the third: 3.3% (95% CI: 2.6, 4.2) versus 1.3% (95% CI: 0.89, 1.7); no difference was observed between the first and second surges. More cases required ventilation during the second surge compared to the first or third: 32.8% (95% CI: 2.2, 3.6) versus 0.6% (95% CI: 0.18, 1.5) and 1.6% (95% CI: 1.2, 2.2), respectively. The mortality rate was higher in the second surge compared to the first and third: 4.8% (95% CI: 4.0, 5.8) versus 1.1% (95% CI: 0.15, 2.2) and 2.4% (95% CI: 1.9, 3.0), respectively, with mortality rates during the first and third surges being similar. The characteristics of the PCC group according to COVID-19 pandemic surges are shown in Table 7.

**Table 7:** Characteristics of PCC based on the COVID-19 pandemic surge.

PCC group	First Alpha pandemic surge		Second Delta pandemic surge		Third Omicron pandemic surge		Omicron variant	
Characteristic	*N* = 120^a^	95% CI^b^	*N* = 211^a^	95% CI^b^	*N* = 280^a^	95% CI^b^	*N* = 654^a^	95% CI^a^
Emergency department visit (yes)	12 (11%)	5.8, 18%	16 (8.0%)	4.8, 13%	17 (6.5%)	4.0, 10%	36 (5.8%)	4.2, 8.0%
Unknown	6		12		19		35	
Primary care visit (yes)	106 (93%)	86, 97%	192 (96%)	93, 98%	251 (96%)	93, 98%	603 (97%)	96, 98%
Unknown	6		12		19		35	
Hospitalization with a COVID-19 diagnosis as the primary diagnosis (yes)	33 (29%)	21, 38%	37 (19%)	14, 25%	44 (17%)	13, 22%	67 (11%)	8.5, 14%
Unknown	6		12		19		35	
Hospitalization where COVID-19 is present on admission (yes)	12 (11%)	5.8, 18%	8 (4.0%)	1.9, 8.1%	11 (4.2%)	2.2, 7.6%	19 (3.1%)	1.9, 4.8%
Unknown	6		12		19		35	
ICU (yes)	3 (2.6%)	0.68, 8.1%	7 (3.5%)	1.6, 7.4%	3 (1.1%)	0.30, 3.6%	13 (2.1%)	1.2, 3.7%
Unknown	6		12		19		35	
Myocarditis (yes)	0 (0%)	0.00, 4.1%	1 (0.5%)	0.03, 3.2%	0 (0%)	0.00, 1.8%	1 (0.2%)	0.01, 1.0%
Unknown	6		12		19		35	
Required a ventilator (yes)	1 (0.8%)	0.04, 5.2%	3 (1.4%)	0.37, 4.5%	4 (1.4%)	0.46, 3.9%	8 (1.2%)	0.57, 2.5%
Death (yes)	8 (6.7%)	3.1, 13%	1		1		26 (4.0%)	2.7, 5.9%
Time until death			10 (4.7%)	2.4, 8.8%	11 (3.9%)	2.1, 7.1%		
0–3 months	2 (25%)	4.5, 64%			1		2 (7.7%)	1.3, 27%
4–6 months	1 (13%)	0.66, 53%	0 (0%)	0.00, 34%			3 (12%)	3.0, 31%
7–12 months	3 (38%)	10, 74%	3 (30%)	8.1, 65%	2 (18%)	3.2, 52%	17 (65%)	44, 82%
13 months+	2 (25%)	4.5, 64%	3 (30%)	8.1, 65%	3 (27%)	7.3, 61%	4 (15%)	5.0, 36%
Unknown	112		4 (40%)	14, 73%	5 (45%)	18, 75%	628	

a *n* (%).

b CI, confidence interval.

The characteristics of PCC patients during the COVID-19 pandemic surges were not significantly different. However, the characteristics of PCC patients differed significantly by ER visit (*p* < 0.001), primary care visit (*p* = 0.002), hospitalization (*p* < 0.001), ICU admission (*p* = 0.02), and death (*p* < 0.001) (Table 5).

## 4 Discussion

Our results showed the substantial burden of acute COVID-19 and post-acute sequelae of SARS-CoV-2 infection (PCC) associated with emergency department (ED) visits, outpatient visits, hospitalizations, deaths, and SARS-CoV-2 reinfection in Bahrain. The magnitude of the acute COVID-19 and PCC burden varied with COVID-19 pandemic surges, showing the highest incidence and mortality rates among the older adult population.

There were more cases of acute COVID-19 and PCC during the Alpha and Delta variants compared to Omicron. This is likely due to low vaccination rates in the initial phases of the pandemic or possibly waning immunity, rather than inherent differences among the variants. The data indicated that the highest incidence of those seeking medical attention for either acute COVID-19 or long COVID-19 occurred in the over-75 age group. Similar to our findings, an analysis of 50 countries showed substantial variation in the incidence of COVID-19, ranging from 0.16 to 82.95 per 100,000 during the Delta period and from 0.03 to 440.88 per 100,000 during the Omicron period (2). These temporal variations in disease incidence highlight the need for active COVID-19 surveillance to accurately assess the burden of the disease across age groups, which is essential for implementing effective prevention and control measures.

The data from healthcare settings align with those from India and Pakistan, where admissions during the first COVID-19 pandemic surge were reported to be higher (3, 4). Additionally, ICU admissions requiring ventilation and average mortality rates were greater during the second COVID-19 pandemic surge (3); however, some reports indicate that deaths were more common in the first surge (4). Furthermore, reports suggest a reduction in ICU admissions and mortality trends during the third COVID-19 pandemic surge compared to the first two (5), with lower illness severity scores observed in the third. However, the need for invasive mechanical ventilation remained high during the Alpha, Delta, and Omicron pandemic surges (6).

There was an increase in PCC in those with other risk factors, those experiencing a second reinfection, and the older age group, consistent with the literature (7). During the Omicron COVID-19 surge, there was a significant rise in PCC among individuals infected with the Omicron variant seeking medical attention in the 75+ age group, reporting an incidence of 992.67 per 100,000, followed by the 65–74 age group at 145.74 per 100,000; however, it has been reported that the incidence of PCC does not vary between variants (8). A systematic review and meta-analysis estimated the global pooled prevalence at 0.43 (95% CI 0.36–0.46), with the regional prevalence in Asia being closest to Bahrain’s estimate of 0.51 (95% CI, 0.37–0.65), suggesting that the incidence in Bahrain was not dissimilar to the one reported in Asia and worldwide (9).

Mortality among the acute COVID-19 population under study indicated an increase during the second and third COVID-19 pandemic surges compared to the first surge. This increase predominantly impacted the 75+ age group, with a significant rise in the 65–74 age group compared to younger populations. In a worldwide review (2), the median case fatality rates were recorded at 8.56 (interquartile range [IQR]: 4.76–18.39) during the Delta period and 3.04 (IQR: 1.87–7.48) during the Omicron period. Furthermore, 47 out of 50 countries reported decreased case fatality rates for the Omicron variant, with the rate ratio ranging from 0.02 (95% CI: 0.01–0.03) in Cambodia to 0.97 (95% CI: 0.87–1.08) in Ireland (2).

For PCC, the mortality rate increased during the second and third COVID-19 pandemic surges compared to the first. Mortality was primarily observed in people aged 75 and older; however, the sample size in this study was too small for an accurate assessment. It is challenging to determine the excess mortality rate for PCC from the literature due to the lack of agreed diagnostic criteria to define the condition (13). Nonetheless, it is acknowledged that there is a significant excess mortality associated with age, male sex, diabetes, chronic kidney disease, and pneumonia, among other factors (14). In the case of acute COVID-19 with the Omicron variant, the mortality rate in the studied population was 6.01 per 100,000, significantly higher in the 65–74 age range at 9.72 per 100,000, but predominantly affecting the 75+ age group with a mortality rate of 532.02 per 100,000. For PCC associated with the Omicron variant, the mortality rate in the studied population was 1.78 per 100,000, with deaths primarily occurring in the 75+ age group, where the mortality rate was significantly higher at 142.74 per 100,000.

The demographic data showed that those with PCC were generally older, with a significantly higher proportion in the 65–74 and 75+ age groups. They were also more likely to be female and of Arab descent. While those with PCC had a higher overall vaccination rate, there was no significant difference in the number of vaccine doses received, with over 70% of the studied population having received three doses.

These findings suggest that women may be at greater risk of developing PCC and that there may be a potential ethnic component, with those of Arabic descent more likely to develop PCC compared to those of Asian origin. This is in line with existing literature showing that women are most vulnerable to COVID-19 infection (10). However, while some studies suggest that vaccination may be protective against PCC (11), its role remains unclear (12).

Those with acute COVID-19 had significantly fewer risk factors compared to those with PCC, who had a higher prevalence of cancer, renal, respiratory, and cardiovascular diseases, diabetes, hypertension, neurological disorders, obesity, and severe mental disorders. Only human immunodeficiency virus (HIV), liver disease, and solid organ transplantation showed no significant differences between the acute COVID-19 and long COVID populations. The association between comorbidities and the risk of long COVID is well recognized (15, 16) and is reflected in the comorbidities reported in this study.

A comparison of healthcare utilization during the COVID-19 pandemic surges showed no significant differences in ED or primary care visits. However, hospitalizations with COVID-19 as the primary diagnosis were higher during the first surge compared to the second and third surges; this trend aligns with the low vaccination rates in the early stages of the pandemic. Additionally, there was an increase in ICU admissions during the second surge compared to the third, along with an increase in the number of patients requiring ventilation during the second surge and a rise in mortality, particularly in the first 3 months of their initial presentation to medical services. These observations may partly result from the initial vaccination strategy using Sinopharm, which is known for having more rapidly waning immunity (17).

A comparison of healthcare utilization during the COVID-19 pandemic surges for PCC showed no differences in ED or primary care visits, nor in hospitalizations where COVID-19 was the primary diagnosis, between the first surge and the second and third surges. There were also no differences in ICU admissions, the number requiring ventilation, or the mortality rate, which remained consistent throughout the study period; however, the number of recorded deaths was too low to allow for accurate interpretation (18).

Bahrain’s vaccination program is recognized for its high coverage, with over 70% of the population receiving three doses of the vaccine. A comparison with advanced countries such as Israel and the United Arab Emirates, which also implemented aggressive vaccination campaigns (19), indicates that new infections and deaths are significantly negatively associated with the proportion of the population vaccinated with at least one dose (13). Depending on the first-dose coverage, a greater proportion vaccinated with two doses appears to provide no further reductions in new cases and deaths (13). However, the effectiveness of the vaccination strategy in preventing long COVID remains unclear (14), particularly in countries facing vaccination disparities (20).

As noted, this study found that individuals with PCC were more likely to be older, female, and of Arab descent. Similar demographic trends have been observed in countries like Italy and Spain (21), where older populations and women have shown higher vulnerability to long COVID. Comparing these findings could help identify universal risk factors and inform targeted interventions. As noted in this study, the burden of COVID-19 in Bahrain varied significantly across different pandemic surges, with higher case numbers during the Alpha and Delta variants compared to Omicron. This pattern is consistent with observations in other countries, such as the United States and the UK, where the impact of different variants has been documented (22). Understanding these trends can provide a broader perspective on how various variants affect public health. Bahrain’s healthcare infrastructure, which facilitated a rapid and effective vaccination rollout, can be compared to countries such as Singapore, which is known for its efficient healthcare systems. Such comparisons could reveal how healthcare infrastructure influences the management and outcomes of COVID-19.

This study on PCC in Bahrain highlights variations in PCC rates across different regions. Several factors could contribute to these differences, including healthcare access, genetic predisposition, and socioeconomic factors (23). Access to healthcare services can significantly influence PCC rates. Regions with robust healthcare systems may manage acute COVID-19 cases better, potentially reducing the severity and incidence of long COVID. Conversely, ethnicity, discrimination, and limited access to healthcare are associated with PCC (24).

The study notes that Bahrain’s healthcare infrastructure played a role in managing COVID-19, which could be compared to other regions with similar systems. Genetic factors might contribute to the susceptibility and severity of PCC. The study indicates that individuals of Arab descent are more likely to develop PCC, suggesting a potential genetic component. This aligns with global research indicating that genetic predispositions can affect immune responses to SARS-CoV-2 (25). Socioeconomic status can impact both the exposure risk to COVID-19 and the ability to access timely medical care. In regions with greater socioeconomic disparities, individuals may face barriers to accessing healthcare, leading to delayed treatment and an increased risk of developing PCC (26). The study’s demographic data suggest that socioeconomic factors may contribute to PCC incidence, as observed in the higher incidence among certain population groups. In addition, industries that involve public interactions, such as teaching and education, social care, healthcare, civil service, retail, and transport, have the greatest likelihood of long COVID (27).

The impact of vaccination on the development of PCC remains uncertain; however, vaccination is suggested to reduce the probability of long COVID symptoms (11). We did not specifically examine different types of vaccines, nor was there evidence of differences in vaccination status between acute COVID-19 and PCC or in the age-stratified number of vaccine doses administered.

While this study examined comorbidities (e.g., diabetes, cardiovascular diseases, obesity), other risk factors such as socioeconomic status and occupation were not often documented in the electronic medical records. This is unfortunate, as research has shown that long COVID disproportionately affects certain patients based on their social determinants of health (23), including healthcare access, socioeconomic status, and occupation, as noted above.

A strength and a limitation of this study was that it was conducted in a well-defined population primarily consisting of Arab cases, with the remainder being long-term residents of Asian descent. One limitation of the study was that it relied on administrative data, which restricted the available information to what was present in the databases and did not capture individuals who did not engage with the national healthcare system, potentially leading to an underestimation of the true burden of the disease. Additionally, genomic sequencing is not routinely performed on all confirmed COVID-19 cases; therefore, the type of variant was inferred based on the date of a positive test and the predominant variant during each COVID-19 pandemic surge in Bahrain at the time of testing.

## 5 Conclusion

The burden of acute COVID-19 and PCC varied substantially across pandemic surges, influenced by community herd immunity and vaccination status. Our findings are consistent with the recommendations of the CDC and WHO, which advocate for early and timely vaccination, particularly for older adult individuals and non-older adult individuals with risk factors, to reduce the risk of complications from SARS-CoV-2 infection. In addition, while our study provides valuable insights into the comorbidities associated with PCC, a comprehensive understanding of PCC risk requires consideration of additional factors such as socioeconomic status, occupation, and healthcare access. These factors not only influence both the likelihood of contracting COVID-19 but also affect the severity of its long-term effects.

## Data Availability

The original contributions presented in the study are included in the article/Supplementary material, further inquiries can be directed to the corresponding author.
