# Supplementary material for: Burden of acute and long-term COVID-19: a nationwide study in Bahrain
Source: Front Public Health. 2025 Mar 18;13:1539453. doi: 10.3389/fpubh.2025.1539453 (PMC11958954; doi:10.3389/fpubh.2025.1539453)

**Table S1**. COVID-19 patients were grouped based on the healthcare settings such as inpatient, outpatient or ICU that they encounter within the 90 days on and following their index COVID-19 diagnosis.

| **Acute COVID status** | **Healthcare setting** | **Data source(s)** | **Outcome definition** |
| --- | --- | --- | --- |
| Outpatient COVID-19 | Outpatient | COVID-19 national surveillance and administrative database | COVID-19 health reimbursement claims in the 90-day evaluation period in the outpatient setting. |
| Inpatient COVID-19 | Inpatient without ICU admission | COVID-19 national surveillance and administrative database | Hospitalization with a COVID-19 diagnosis as the primary diagnosis or where COVID-19 is present on admission (POA) **with no** evidence of an intensive care unit (ICU) stay occurring during the 90-day evaluation period. |
| ICU COVID-19 | ICU | COVID-19 national surveillance and administrative database | Hospitalization with a COVID-19 diagnosis as the primary diagnosis or where COVID-19 is present on admission (POA) **with** evidence of an intensive care unit (ICU) stay occurring during the 90-day evaluation period. |
| Inpatient COVID-19 related death | Inpatient | COVID-19 national surveillance and administrative database | Death occurred during hospitalization with laboratory confirmed COVID-19 at the point of hospital admission. |
| COVID-19 related death outside hospital | Non-inpatient | Administrative database | COVID-related death occurred outside hospital in the 90 days following laboratory confirmed COVID-19 diagnosis. |
| Myocarditis and Pericarditis | Inpatient and outpatient | Administrative database | Myocarditis and/or pericarditis occurred during 90 day evaluation period. |
| Acute kidney disease | Inpatient | Administrative database | Acute kidney disease occurred during the 90 day evaluation period. |

**Table S2**. The ICD10 codes were used to determine the burden of PCC complications.

| **Category** | **Condition** | **ICD10 codes** |
| --- | --- | --- |
| Cancer | Cancer | All cancer ICD codes  Chemotherapy: CPT J9000 – J9999; ICD10: Z51.11 |
| Cardiovascular disease | • Cardiac failure  • Cardiomyopathy  • Dysrhythmias  • Peripheral arterial disease  • Coronary artery disease  • Cerebrovascular disease (including stroke) | I50  I42.9  I49  I73.9  I20.X, I23.X, I24.X, I25.X  I60-I69 |
| Chronic renal disease | Chronic renal disease | N18 |
| Chronic respiratory disease | • Asthma  • COPD  • Bronchiectasis | J45.909  J44.9  J47 |
| Diabetes Mellitus | • Diabetes Mellitus 1  • Diabetes Mellitus 2 | E08-E13 |
| HIV | • HIV | B97.35, Z21 |
| Hypertension | • Hypertension | H35031, H35032, H35033, H35039, I10, I110,  I119, I120, I129, I130, I1310, I1311, I132, I150, I151,  I152, I158, I159, I674, N262 |
| Liver disease | • Alcoholic liver disease  • Fatty liver disease  • Cirrhosis | K70.9  K76.0  K74.60 |
| Neurological disorder | • Epilepsy  • Parkinson’s disease  • Dementia (any cause, including Alzheimer’s disease) | G40  G20  G30.0, G30.1. G31.83  F05, F02.81, F02.80, 291.83 |
| Overweight/obesity | • BMI >25 | E66  Z68.XX |
| Severe mental disorder | • Bipolar mood disorder  •Schizophrenia | F31  F20.9 |
| Solid organ transplant | • History of Kidney, liver, heart, or lung transplant | Z94.X |

**Table S3.** A list of medical conditions that were considered for PCC that were identified using the ICD10 codes listed in Supplementary table 2, which were used to determine the burden of PCC.

| **Potential post-COVID complications** | **Conditions** |
| --- | --- |
| Cardio-vascular disease complications | Cardiac failure, cardiomyopathy, dysrhythmias, peripheral arterial disease, coronary artery disease, cerebrovascular disease (including stroke) |
| Respiratory complications | Pneumonia, asthma, exacerbation of COPD |
| Liver disease complications | Non Hepatitis B and C, non-alcoholic liver disease, cirrhosis, liver transplant |
| Chronic kidney disease complications | Chronic kidney disease stage I, II, III, IV, V |
| Neurological complications | Stroke, ischaemic stroke, haemorrhagic stroke, cerebrovascular accident, transient ischaemic attack, intracerebral haemorrhage, intracranial haemorrhagic, cerebral venous sinus thrombosis |
| Diabetes | Diabetes type I and II |
| Thrombosis | Pulmonary embolism, deep venous thrombosis, venous thromboembolism, hepatic vein thrombosis, Budd Chiari syndrome, mesenteric vein thrombosis |
| Multisystem inflammatory syndrome | Inflammation of heart, lungs, kidneys, brain, skin, eyes or gastrointestinal organs* |

*CDC definition will be used: <https://www.cdc.gov/mis/mis-c/hcp/index.html>

**Supplementary figure 1.** Timeline of the COVID-19 vaccination program in the Kingdom of Bahrain. EUA=emergency use authorization; SII=Serum Institute of India. Source :[2]
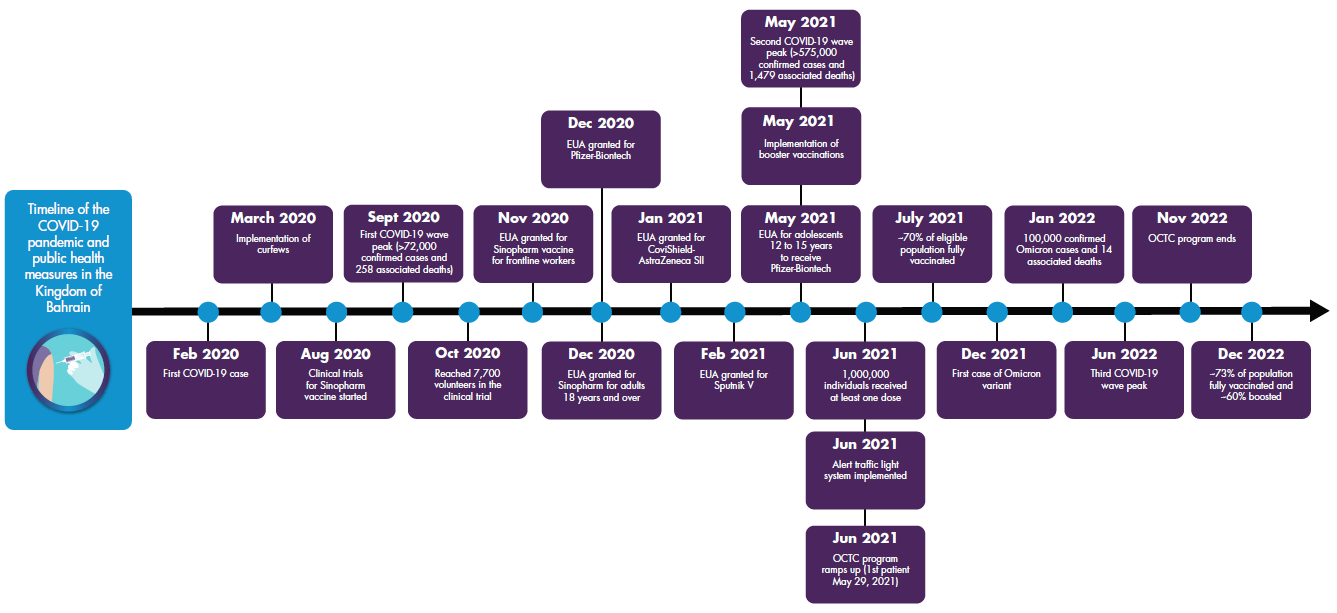


**Supplementary figure 2.** Monthly pattern of sequenced lineages in the Kingdom of Bahrain


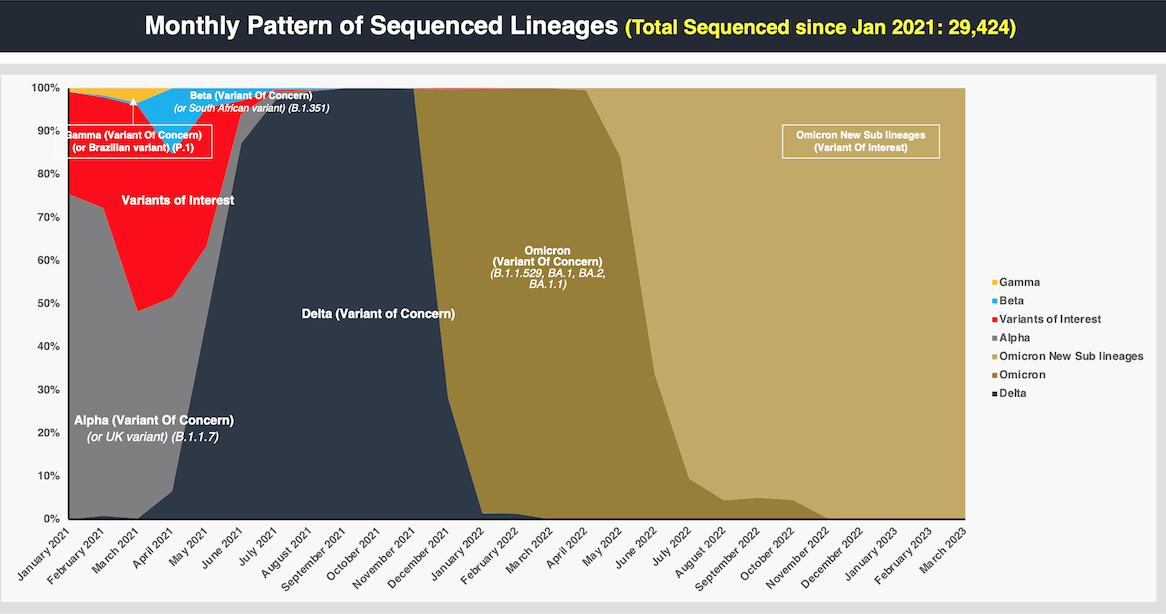

Supplement: Supplementary file 1 [file Supplementary_file_1.docx]
